# Dispatcher nurses’ experiences of handling drones equipped with automated external defibrillators in suspected out-of-hospital cardiac arrest - a qualitative study

**DOI:** 10.1186/s13049-024-01246-6

**Published:** 2024-08-21

**Authors:** Dalby-Pedersen Hanna, Bergström Erika, Berglund Ellinor, Schierbeck Sofia, Svensson Leif, Nord Anette, Hollenberg Jacob, Claesson Andreas

**Affiliations:** 1 Emergency Medical Services, Sjukhusen i Väster, Region Västra Götaland, Dumpergatan 3, Kungälv, Kungälv, 442 40 Sweden; 2 Emergency Medical Services, Premedic Ånge, Region Västernorrland, Spångbrovägen 1, Ånge, 841 32 Sweden; 3 Centre for Resuscitation Science, Department of Clinical Science and Education, Karolinska Institutet, Sjukhusbacken 10, Södersjukhuset, Stockholm, S-118 83 Sweden; 4 Department of Medicine, Karolinska Institutet, Solna, Stockholm, S-171 77 Sweden

**Keywords:** Automated external defibrillator (AED), Cardiopulmonary resuscitation (CPR), Dispatch centre, Drone, Emergency medical dispatch centre (EMDC), Out-of-hospital cardiac arrest (OHCA), Unmanned aerial vehicle (UAV)

## Abstract

**Background:**

Reducing the time to treatment by means of cardiopulmonary resuscitation (CPR) and defibrillation is essential to increasing survival after cardiac arrest. A novel method of dispatching drones for delivery of automated external defibrillators (AEDs) to the site of a suspected out-of-hospital cardiac arrest (OHCA) has been shown to be feasible, with the potential to shorten response times compared with the emergency medical services. However, little is known of dispatchers’ experiences of using this novel methodology.

**Methods:**

A qualitative semi-structured interview study with a phenomenological approach was used. Ten registered nurses employed at an emergency medical dispatch centre in Gothenburg, Sweden, were interviewed and the data was analysed by qualitative content analysis. The purpose was to explore dispatcher nurses’ experiences of deliveries of AEDs by drones in cases of suspected OHCA.

**Results:**

Three categories were formed. Nurses expressed varying compliance to the telephone-assisted protocol for dispatch of AED-equipped drones. They experienced uncertainty as to how long would be an acceptable interruption from the CPR protocol in order to retrieve a drone-delivered AED. The majority experienced that collegial support was important. Technical support, routines and training need to be improved to further optimise action in cases of drone-delivered AEDs handled by dispatcher nurses.

**Conclusions:**

Although telephone-assisted routines for drone dispatch in cases of OHCA were available, their use was rare. Registered nurses showed variable degrees of understanding of how to comply with these protocols. Collegial and technical support was considered important, alongside routines and training, which need to be improved to further support bystander use of drone-delivered AEDs. As the possibilities of using drones to deliver AEDs in cases of OHCA are explored more extensively globally, there is a good possibility that this study could be of benefit to other nations implementing similar methods. We present concrete aspects that are important to take into consideration when implementing this kind of methodology at dispatch centres.

**Supplementary Information:**

The online version contains supplementary material available at 10.1186/s13049-024-01246-6.

## Introduction

Sudden out-of-hospital cardiac arrest (OHCA) is a major health problem associated with a high mortality rate in Europe. Out of about 275,000 cases, the survival rate is 10.7% [1]. Similar survival rates are found in Sweden, where 10.8% of approximately 6,000 cases survived for a period of more than 30 days after the arrest [2]. When a victim suffers from OHCA, several aspects of the situation must work optimally to increase the chances of survival. As described by The European Resuscitation Council regarding the “chain of survival”, these aspects can be divided into four steps: early recognition and call for help, early bystander cardiopulmonary resuscitation (CPR), early defibrillation and early advanced life support, and standardized post-resuscitation care [3]. Chances of survival may increase by up to 50–70% if the heart is defibrillated within three to five minutes after a cardiac arrest [3].

### Background

As ambulance response times in Sweden have increased [2] and the majority of cardiac arrests occur in people’s homes [4, 5], it is important to find alternative strategies to reduce the time to treatment with an automated external defibrillator (AED). One such novel method is the use of unmanned aerial vehicles (UAVs), i.e., drones, to deliver AEDs [6]. Geographical information system analysis (GIS) has been successful when calculating optimal locations for UAVs [7]. Hypothetically, UAVs can deliver AEDs in rural areas before ambulances in 93% of cases, saving an average of 19 min per case [8]. The feasibility of AED delivery by UAVs has also been investigated in highly populated areas, showing that UAVs can deliver AEDs before the arrival of emergency personnel in 26% of cases, saving an average of three minutes per case [9]. This demonstrates that UAVs may contribute by shortening the time to defibrillation in both urban and rural environments.

The dispatch of UAVs for delivering AEDs in real-life cases of OHCA has been shown to be feasible, and a UAV-delivered AED was used successfully in a case of OHCA in 2021 [6, 10, 11]. Use of UAVs may play an essential role as a tool to further develop the chain of survival [12]. Simulation studies have been carried out to investigate bystander experiences of handling a UAV-delivered AED [13, 14], but to the best of our knowledge, the experiences of dispatch personnel when using and interacting with this novel system have not previously been explored. Thus, such studies are important in the development and improvement of this novel methodology, and the findings may also have international impact when considering nations that are launching similar methods.

The purpose of this study was to explore the experiences of dispatch personnel (registered nurses) in delivery of AEDs by UAVs in cases of suspected OHCA.

## Methods

This is a qualitative study using a phenomenological approach to explore the perception and understanding of dispatchers’ experiences at an emergency medical dispatch centre (EMDC) when activating a novel system using UAVs to deliver AEDs in cases of suspected OHCA. This approach was aimed at investigating real experiences, and according to Polit and Beck [15], is useful when a phenomenon has been poorly defined.

### Context

Emergency medical services (EMS) may be reached via the Primary Service Answering Point by dialling the national telephone number 112, and in the region of Västra Götaland, there are about 110 ambulances that cover about 1.7 million inhabitants.

Dispatch of three AED-equipped UAVs was initiated on June 1st, 2020 over a 4-month period in a feasibility study covering an estimated 80,000 inhabitants in the region of Västra Götaland, Sweden [6]. Follow-up studies have been ongoing with five UAVs since April 21st 2021. Registered nurses (RNs) at the EMDC are instructed to refer callers to retrieve a UAV-delivered AED in cases where the UAV arrives before the EMS, and they have access to an AED and carrying case to touch and feel the device that bystanders encounter. Due to a heavy workload at the EMDC during initiation of the research project, the focus on training and information on the drone project for RNs successively increased from 2020 to 2023. When the study was conducted, the UAVs had delivered AEDs 47 times.

### Sampling strategy and units of study

Purposive selection was used to ensure that the informants had experience of the phenomenon under investigation [15]. Since the dispatch of UAVs with AEDs is implemented at only one EMDC in Sweden, there were a limited number of potential informants available for the purpose of this study. Selected participants for interviews were registered nurses, with or without specialist training, who had handled at least one call where a UAV had been dispatched with an AED to the location of an OHCA. Ten out of 15 possible informants participated; they were evenly divided in gender and were between 31 and 53 years old. Overall, they had clinical experience as RNs ranging from six to 27 years.

### Researcher reflexivity

The two interviewers were RN paramedics, and therefore, had pre-understanding of how to deal with different new situations arising with drones and the complexity of OHCA situations, and interactions with bystanders, relatives and the EMDC. Throughout the whole process these difficulties were raised among the authors in order to avoid potential result bias. One of the two interviewers was acquainted with two participants, but this was not considered to have affected the results, as in these two cases the interviews were led by the other one.

### Data collection, analysis, instruments and methods

Semi-structured interviews were conducted after purposive selection of ten participating dispatchers, all RNs. This interview technique is the most commonly used method when collecting data for qualitative research [16] and was therefore considered appropriate. The use of content analysis using coding of the written dataset derived from interviews was considered favourable in comparison with a thematic analytic model, as the researchers had preexisting ideas and pre-understanding of the context. This inductive approach generalizes from what was observed and is associated with previous experiences. All interview sessions were led by two of the authors, Dalby-Pedersen and Bergström. In order to create as much openness as possible as regards the informants’ experiences, and to minimise the risk that the interviewers’ pre-understanding would colour and control the results [17], a single, broad and open question was used; “*Can you tell us about your experiences regarding cardiac arrest cases*,* where the UAV has flown towards the address*,* from the beginning to the end of the call?”.* Follow-up questions such as “Can you develop?”, and “Can you explain?” were used when clarification was needed.

Data collection was conducted over a period of two weeks in March 2022. Nine of the ten interviews were conducted via Zoom, i.e., in an online video and audio meeting room, and one interview took place at the EMDC. The use of Zoom meetings in qualitative research is considered a beneficial approach in data collection [18] and was suitable for use in connection with the ongoing Covid-19 pandemic.

The interviews varied in duration between 10 and 35 min, and the conversations, i.e., voice logs, were recorded and afterwards transcribed into text in Microsoft Word. Deidentification of the participants was carried out, and the transcripts were kept in code-locked computers. The contents of the interviews were kept classified during the process. The text was analysed by using content analysis, as described by Elo and Kyngäs [19] (example in Table 1). Similar meaning-bearing units were compiled and encoded. Codes that concerned the same aspects were placed under a common subcategory, and similar subcategories formed categories. During the analytic process, several categories were noted that were then excluded, as they did not answer the purpose of the study. Standards for reporting qualitative research [20] were used, please see supplement 1.

**Table 1:** Example of content analysis methodology

Headings from the transcripted interview	Meaning-bearing unit	Code	Sub category	Category
“*but if they are by themselves*,* they should not interrupt compressions*”	“they should not interrupt compressions”	Interruption of CPR	Uncertainty regarding how long compressions can be paused	The RNs’ emotions and uncertainties in guiding the bystander to use the AED delivered by UAV

*From the transcriptions derived from voice logs of the RNs’ experiences. Voice logs were extracted and condensed in a stepwise manner from headings into meaning-bearing units and categories.*

## Results

During analysis of the transcribed material, eight subcategories and three categories were formed.

### Category 1. The RNs’ emotions and uncertainties in guiding the bystander to use the AED delivered by a UAV

This category describes how the nurses at the EMDC experienced handling cases of suspected cardiac arrest. Stress and tunnel vision among the nurses were highlighted as obstacles when handling these cases in an efficient and patient-safe manner without losing focus on the task. The category highlights uncertainty about when it is appropriate to interrupt compressions during cardiopulmonary resuscitation.

#### Psychological factors that affect RNs’ work

The informants described that perceiving a situation as difficult, or becoming frustrated by not being able to persuade bystanders to start CPR, negatively affected their work effort. Stress was raised as a risk of negatively affecting their ability to guide the bystander in telephone-assisted CPR (T-CPR). Some informants highlighted the fact that when they were focused on T-CPR and the bystander, they easily ended up with tunnel vision, which led to difficulties in paying attention to the UAV’s activity on the computer screen.

*“…you are stressed and influenced by the conversation.” I. 4*.

Feelings of satisfaction, trust in the bystander’s competence and positive feelings were also described. Some informants described that it was a relief and very valuable to have a UAV delivering the AED, and that it was satisfactory on the occasions when it was used.

*“It was fantastic! It was as if the conditions for the patient could not have been better.” I. 9*.

#### Uncertainty regarding how long compressions can be paused

There were different views among the informants regarding how long they could ask the bystander to interrupt CPR to retrieve the AED. Some informants expressed the fact that they had guidelines concerning when and how to ask the bystander to interrupt compressions, but they were not familiar with them. Some informants believed that it was not practical to interrupt CPR to retrieve the AED if there was a lone bystander at the scene. Some informants stated that they chose to refer to the AED even if the bystander was alone at the scene. According to the informants, the decision to refer depended on how long it would take for an ambulance or other resource to arrive at the scene. The majority of informants described the importance of shortening the time to the first defibrillation and that an interruption in compressions of a few minutes was felt to be reasonable.

*“I feel that a 2- to 3-minute break is reasonable in any case to pick up a AED.” I. 10*.

*“But I had a case where he was alone. So he could not run out and get the AED because he couldn’t interrupt CPR… and I did not want that either.” I. 7*.

Some informants described that they did not want to ask bystanders to pause compressions even though the UAV had delivered the AED before an ambulance or other resource arrived at the scene. The view was that you should not interrupt ongoing compressions. Concern that it would take too long to instruct the bystander in use of the AED was stated by one informant as a reason why the focus was on the bystander continuing with compressions.

*“If I’m going to instruct him on how to open the hatch and… like that would take a long time and you wanted to continue… with the compressions anyway.” I. 7*.

*“You should not pause in a CPR situation.” I. 5*.

### Category 2. Challenges and conditions that affect the use of UAV-delivered AEDs

This category covers characteristics of bystanders in the practice of CPR. The physical limitations of bystanders and their willingness to carry out instructions were reasons that influenced whether or not a UAV-delivered AED was used. The category also covers the importance of good communication with the bystander. Further, the category covers how positioning of other first responders were factors that affected whether or not the bystander was referred to a UAV-delivered AED.

#### Physical limitations and psychological aspects of bystanders

The informants described different types of physical challenges for bystanders in performing CPR. They considered that referral to a UAV-delivered AED was not a priority, as CPR was perceived to be challenging for the bystander, particularly when it was suspected that their physical limitations would be an obstacle to retrieval of an AED.

*“Then at some point I knew it was an elderly person who was doing it*,* where you could barely get her to understand and do CPR and then I thought*,* how are you going to get her to pick up an AED and be able to use it?” I. 2*.

It was also described that psychological factors could be an obstacle for the bystander. One informant described that when a bystander was in shock, this led to mental locking, where the bystander only stood and screamed and was unable to act. Stress in bystanders was raised as a difficulty in guidance of CPR and referral to AEDs. One informant highlighted the fact that in some cases, CPR is the only thing that bystanders are able to perform, without other distracting tasks.

*“And to me it feels like*,* if I give him another assignment now… then maybe he’ll lose it a little bit.” I. 7*.

Factors such as the bystander’s perception that the patient was already deceased, or fear of approaching an unknown person were further described. One informant mentioned that in some cases it was only possible to gently encourage the start of CPR, and thus not possible to force the bystander to perform CPR or retrieve the drone-delivered AED if they did not want to. The informants also had different experiences of how communication with bystanders worked. Some bystanders needed very detailed guidance, while others barely needed assistance from the RNs. If bystanders and the RNs were calm and could communicate clearly, and there were several bystanders on site, retrieval and use of a drone-delivered AED was successful to a greater extent. Some informants mentioned that it was not possible to interact with the bystander, as the stress reaction to the situation made the possibility of good communication difficult. The informant said that in such a situation it was important to be there as support on the ’phone. Another informant felt that it was a good dialogue even though the bystander could not be persuaded to start CPR.

*“I’m trying to get him to start CPR*,* but he won’t do it because he thinks she’s dead… No*,* but it still felt like it was a good conversation with them.” I. 4*.

#### Challenges and prerequisites for referral to and handling of an AED

The number of bystanders, the exact location of the patient and the mobility of the bystanders were highlighted as important pieces of information for the RNs in order to determine the feasibility of AED referral. It was described that both the UAV and the AED could be heard clearly on arrival, and there were no difficulties in detecting or referring callers to it. It was also perceived that the AED was easy to use even in stressful situations. The majority of informants found it easy to instruct and easy for the bystander to connect the AED to the patient once it had been retrieved. But uncertainty was also expressed about how the AED should be disconnected from the UAV.

*“I don’t know how the AED is disconnected from the drone and how they are going to use it.” I. 4*.

*“Then you hear it start beeping*,* so it was super easy. After all*,* it only took a few seconds from the time it arrived to the time it was somehow in place…” I. 9*.

The majority of informants believed that the outcome for the patient depended on the number of bystanders at the scene, and their characteristics. One informant described that it could be chaotic at the site if there were several bystanders, while in other cases everything had worked out well. In cases where the bystanders had been healthcare personnel or mobile ’phone-positioned volunteer first responders with CPR training, it was described that referral to and handling of the AED had worked well.

#### Impact of different first responders on UAV handling by RNs

The informants described that it was not always deemed appropriate to refer to the AED. One reason was that the UAV did not always arrive first at the scene, and if another first responder already was or soon would be at the scene, the informant prioritised continuation of compressions instead of interruption of CPR in order to retrieve the AED. An informant said that he had cancelled the UAV when the ambulance and rescue services were about to arrive at the scene:

*“On a few occasions*,* I have cancelled the drone as I saw no benefit [of having] an extra AED*,* as the ambulance and emergency services soon would be on the scene.” I.1*.

Some informants said that because UAVs deliver AEDs in ambulance-dense and relatively densely populated areas, other AEDs usually arrive before the UAVs. However, some experiences were that when a UAV-delivered AED was the first on site, and where this was felt to be a time gain, it would be best to refer the bystander to it.

### Category 3. Tools that facilitate and affect the RNs’ work effort

This category highlights the importance of clear communication and well-functioning collaboration between colleagues at the EMDC. The category also highlights experiences and challenges that the participants experienced when using technical equipment. Also described are strengths and limitations that are experienced, as well as the use of flowcharts, routines and training needs.

#### Peer support

Collegial support in verbal and written form was described by the majority of informants as a great help in their handling of a case of cardiac arrest. The informants felt that colleagues at the EMDC could be helpful by listening in on the call, and that they alerted the RNs when the UAV had delivered the AED and when referral was possible. It was perceived as good to have collegial cooperation in those cases in which it is difficult to have full attention to all the details. Some informants had only received written support in a chat function and felt this was sufficient, while others also wanted verbal support. Some informants had only received verbal support and wanted written support as a supplement, but the majority of informants felt that the collaboration worked excellently. When the informants received support, they felt that they could more easily have an overview of all aspects of the case. Therefore, it was felt to be a great advantage to have two RNs working together.

*“Our helicopter emergency medical service coordinators are very good at stepping in and saying the right things at the right times.” I. 9*.

One informant felt that sometimes it was perfectly reasonable to be alone when handling the case. In such a situation the process went smoothly, as there were several bystanders on site who were trained in CPR. The UAV delivered the AED to the location of the patient, and mobile-positioned volunteer first responders arrived on the scene soon afterwards. Another informant said that according to operational routine, the drone operator calls the operational management team at the EMDC and informs them of where the AED has been delivered, but it was important that the information also came in writing onto their computer screens.

*“…the section leader may not have the ability to take that exact call. They have other things to do too. So it’s not certain that you can answer the call immediately.” I. 1*.

#### Technical support and difficulties

The informants mentioned that they have a chat function where they receive written support from the Helicopter Emergency Medical Service coordinator regarding, among other things, the position of the UAV. Some informants experienced this type of support as being clear, while others felt that it was difficult to pay attention, as there is a lot of other activity on the computer screen at the same time. The informants said that when the UAV was activated, this information appeared in a tab on the computer screen. Several informants stated that the UAV tab was clearly visible on the screen, while some felt that the UAV’s position during the case could be more distinct. Difficulties with not being able to follow the UAV’s position in real time was also mentioned, and this impaired interaction with the bystanders.

*“It comes up as a tab like all other resources*,* including the ambulance and the rescue service*,* and the police vehicles as well. So*,* it becomes one in the crowd…” I. 10*.

#### Inadequate routines and training

It was mentioned that the RNs have a flowchart with pictures and text to follow as an aid in communication with the bystander. However, problems were reported concerning the flowchart currently having an unclear layout that was difficult to follow. Another informant reported that even when the flowchart was available, it was not used.

*“We also have very good instructions that we must follow*,* exactly how you should say*,* so that we don’t say different things*,* so there will be simple instructions for the caller.” I. 8*.

One informant emphasised that it was up to each individual nurse to take responsibility for their own learning regarding UAV function and to familiarise themselves with the routines. It was found that some were more likely to seek information than others, resulting in the former being more comfortable using the resource and referring to the AED than those who were not as knowledgeable. Some informants felt that there was an expectation that they should be able to handle the AED, as well as able to explain to a bystander how it should be used. They also experienced a need for training in CPR, with a desire for scenario training focusing on technical handling and practical handling of the AED to improve the management of these cases.

*“So*,* I can feel that we would have needed… CPR training to make it work a little better.” I. 9*.

Several informants mentioned that the function of UAVs delivering AEDs is relatively new and therefore not implemented into their way of working. Several informants stated that there is a need for more extensive training for the resource to be used optimally.

*“It takes training to get it right and completely understand when the drone has arrived.” I.6*.

## Discussion

The main finding in this study was that RNs had a heterogeneous understanding of how to comply with the T-CPR protocol when drones were dispatched to cases of suspected OHCA. In the first category it was highlighted that the informants experienced stress and frustration when they failed to establish contact with bystanders. These findings can be compared with those in previous research, indicating that nurses who work in an EMDC find it emotionally challenging to handle life-threatening cases, while others do not feel that they are affected [21]. Similar findings were highlighted by Gustafsson and Eriksson [22], who discovered that nurses who worked in telephone counselling experienced anxiety and fear of making wrong judgments, and therefore performing poorer work when they were stressed and tired. Allan et al. [23] found that stress in nurses working on telephone helplines was associated with cognitive failures that may lead to a higher risk of misjudgement and therefore poorer patient outcomes. Future studies are needed on how symptoms such as stress, anxiety and tiredness could be alleviated in order to facilitate nurses’ work, and in the long run, better patient outcomes. In the current study, the informants experienced that stress aggravated their ability to pay attention to the UAV’s activity, highlighting the importance of the fact that the RNs should be capable of handling stress in order to induce bystanders to use UAV-delivered AEDs. Furthermore, the informants were unsure about how long an acceptable interruption of CPR should last in order to retrieve an AED. Several studies have emphasised the importance of early defibrillation [3, 24–28], but they have also highlighted the importance of continuous chest compressions during CPR [12, 29, 30]. Because of this it can be speculated that the RNs had difficulty asking bystanders to pause compressions even though an AED could be retrieved if it was nearby and easily accessible [31]. Early bystander defibrillation is associated with an increased chance of survival [13, 32, 33], and a significantly higher survival rate has been noted when early defibrillation is performed by a bystander, versus those who wait for prehospital personnel [32]. This emphasises the importance of UAV-delivered AEDs being used in cases when arriving first on scene. Drone-delivered AEDs might also come into greater use if dispatched in areas with prolonged EMS response times.

In the second category of the results, the informants described various reasons for choosing not to refer to the AED. Despite a continuous e-learning programme, the presence of a study protocol for T-CPR during dispatch of AED-equipped UAVs with text-lines to be read and used, information at each workstation, and an AED for “touch and feel” to support referral of callers to UAV-delivered AEDs, RNs experienced a need for clearer guidelines. The findings are in line with those of Fredman et al. [25], who reported that lone bystanders were not referred to nearby public AEDs. In this study, informants experienced difficulties in referring bystanders to the AED when they were elderly and alone at the scene, which in many cases meant physical difficulties in performing CPR. Similar findings were made by Sanfridsson et al. [13], who noted that CPR is physically demanding. This implies that referral to an AED should occur when there are at least two bystanders on site. Further, difficulties regarding communication with bystanders were described. Stress and shock in the bystanders significantly impaired their ability to assimilate the RNs’ instructions and to act on them. The results of the current study can be compared with those reported by Fredman et al. [25], who demonstrated that in those cases where the bystander was stressed or had difficulty understanding the spoken language, the likelihood of referral to a public AED decreased. This highlights the challenge for an RN to get a bystander to embrace and act on given instructions. The current results show that the informants felt it was important to function as a support in conversations with bystanders, and similar findings were made by Kaminsky et al. [34]. In the current study, informants described difficulties in communicating with bystanders who were in shock. According to Perkins et al. [35], communication and cooperation as well as the bystander’s awareness of the critical situation are important factors that increase the chances of good cooperation in a life-threatening situation.

In the third category, some informants reported that they felt a need for a more distinct tab for the UAV on the computer screen. This could be done by centring and enlarging the status of the UAV. Further, the informants wanted training in the handling of the UAV-delivered AEDs. This is reinforced by Bergs et al. [36] who found in their study that it is important that personnel receive the necessary education and training when new guidelines are to be implemented. Furthermore, Golding et al. [37] found that a lack of satisfactory training increases stress. This leads to the assumption that adequate training may facilitate the possibility that the RNs’ cognitive load decreases, and that they feel more confident in handling cases where UAVs are involved. Beyond this, the informants felt a need for a clearer layout of the flowchart of AED-drone use, and that it would need to be clarified with larger text and images. The informants work in an environment with a high cognitive load where they use several senses at the same time, i.e., collecting information about the case, instructing bystanders in T-CPR, referring to the AED, monitoring and handling computer screens, and collaborating with colleagues. Working in demanding environments with possible psychological negative effects implies that the implementation of cognitive load theory could be helpful in improve their mental health [37]. The findings are also in line with the results of previous research which shows that the layout of guidelines affects compliance to use it [31]. The use of clear guidelines, i.e., cognitive aids, has been found to contribute to enhanced work effort during high workload and dealing with time-critical tasks [38]. Further, there was an opinion that more dialogue at the EMDC about cases where the UAV had been active would remind the RNs of the possibility of using the resource. This was considered to reduce the risk that the UAV was overlooked in cases of OHCA.

### Limitations

The method of using video-interviews may have both hampered or increased the participants’ willingness to share their experiences. The quality of the conversation and underlying communication, feelings of comfort during the interview and the effects of other factors associated with the video-call, as opposed to a real-life meeting, are currently unknown and may have changed the results if carried out differently. The length of the interviews can be questioned (10–35 min), as it may have influenced the saturation of results. After the interviews were completed, it was considered that several open questions should have been used, as the interviewers perceived the technique as being difficult. As new experiences were presented during the analysis in the last interview, and because the study is limited by the small sample of informants, whether or not the current study has reached saturation cannot be determined (44, 45).

The interviews have not been reviewed by peer debriefing, which could be considered a weakness, as it was the first time the authors analysed interviews and performed a qualitative study. It is questionable and difficult to claim transferability, as the RNs at the EMDC in question are the only ones involved in cases involving UAV-delivered AEDs, but it is possible to assume that the findings could be transferable if this novel methodology is expanded to other EMDCs.

## Conclusions

Although telephone-assisted cardiopulmonary resuscitation routines for drone dispatch were available, the use of these was a rare occurrence. Registered nurses expressed heterogeneous understanding of how to comply with the telephone-assisted cardiopulmonary resuscitation protocol when drones were dispatched. Collegial and technical support was considered important alongside routines and training, which needs to be improved to further support bystander use of drone-delivered AEDs. As the possibilities of using unmanned aerial vehicles to deliver automated external defibrillators in cases of out-of-hospital cardiac arrest are explored more extensively globally, there is a possibility that this study could be of benefit for other nations implementing similar methods. We present concrete aspects that are important to take into consideration when implementing this kind of methodology at dispatch centres.

### Electronic supplementary material

Below is the link to the electronic supplementary material.

Supplementary Material 1

## Data Availability

The datasets used and/or analysed during the current study are available from the corresponding author on reasonable request.

## Abbreviations

AED: Automated External Defibrillator
CPR: Cardiopulmonary Resuscitation
EMDC: Emergency Medical Dispatch Centre
EMS: Emergency Medical Services
OHCA: Out-of-Hospital Cardiac Arrest
T: CPR-Telephone-Assisted Cardiopulmonary Resuscitation
UAV: Unmanned Aerial Vehicle

## References

[1] Gräsner JT, Lefering R, Koster RW, Masterson S, Böttiger BW, Herlitz J, et al. EuReCa ONE-27 nations, ONE Europe, ONE Registry: a prospective one month analysis of out-of-hospital cardiac arrest outcomes in 27 countries in Europe. Resuscitation. 2016;105(2016–08–01):188–95. 10.1016/j.resuscitation.2016.06.004. 27321577 10.1016/j.resuscitation.2016.06.004

[2] Swedish council for cardiopulmonary resuscitation. Annual report Swedish register for cardiopulmonary resuscitation 2021 [Internet]. 2022. https://arsrapporter.registercentrum.se/shlr/20221006/

[3] Semerano F, Grief R, Böttiger BW, Burkart R, Cimpoesu D, Georgiou M, et al. European Resuscitation Council guidelines 2021: systems saving lives. Resuscitation. 2021;161(2021):80–97. 10.1016/j.resuscitation.2021.02.008. 33773834 10.1016/j.resuscitation.2021.02.008

[4] Fredman D, Haas J, Ban Y, Jonsson M, Svensson L, Djarv T, et al. Use of a geographic information system to identify differences in automated external defibrillator installation in urban areas with similar incidence of public out-of-hospital cardiac arrest: a retrospective registry-based study. BMJ Open. 2017;7(5). 10.1136/bmjopen-2016-014801. 10.1136/bmjopen-2016-014801 PMC5623355 28576894

[5] Ovinga I, de Graaf C, Karlsson L, Jonsson M, Kramer-Johansen J, Berglund E, et al. Occurrence of shockable rhythm in out-of-hospital cardiac arrest over time: a report from the COSTA group. Resuscitation. 2020;151:67–74. 10.1016/j.resuscitation.2020.03.014. 2020-06-01. 32278017 10.1016/j.resuscitation.2020.03.014

[6] Schierbeck S, Hollenberg J, Nord A, Svensson L, Nordberg P, Ringh M, et al. Automated external defibrillators delivered by drones to patients with suspected out-of-hospital cardiac arrest. Eur Heart J. 2021;43(15):1478–87. 10.1093/eurheartj/ehab498. 10.1093/eurheartj/ehab498 34438449

[7] Schierbeck S, Nord A, Svensson L, Rawshani A, Hollenberg J, Ringh M, et al. National coverage of out-of-hospital cardiac arrests using automated external defibrillator-equipped drones - a geographical information system analysis. Resuscitation. 2021;163(2021–06–01):136–45. 10.1016/j.resuscitation.2021.02.040. 33675868 10.1016/j.resuscitation.2021.02.040

[8] Claesson A, Fredman D, Svensson L, Ringh M, Hollenberg J, Nordberg P, et al. Unmanned aerial vehicles (drones) in out-of-hospital-cardiac-arrest. Scand J Trauma Resusc Emerg Med. 2016;24(1). 10.1186/s13049-016-0313-5. 10.1186/s13049-016-0313-5 PMC5059909 27729058

[9] Derkenne C, Jost D, De L’Espinay AM, Corpet P, Frattini B, Hong V, et al. Automatic external defibrillator provided by unmanned aerial vehicle (drone) in Greater Paris: a real world-based simulation. Resuscitation. 2021;162(2021–05–01):259–65. 33766669 10.1016/j.resuscitation.2021.03.012

[10] Cheskes S, McLeod SL, Nolan M, Snobelen P, Vaillancourt C, Brooks SC, et al. Improving Access to Automated External defibrillators in Rural and remote settings: a drone delivery feasibility study. J Am Heart Assoc. 2020;9(14):e016687. 10.1161/JAHA.120.016687. 32627636 10.1161/JAHA.120.016687 PMC7660725

[11] Schierbeck S, Svensson L, Claesson A. Use of a drone-delivered Automated External Defibrillator in an out-of-hospital cardiac arrest. N Engl J Med. 2022;386:1953–54. 10.1056/NEJMc2200833. 35584161 10.1056/NEJMc2200833

[12] Olasveengen TM, Semeraro F, Ristagno G, Castrene M, Handleyf A, Kuzovlevg A, et al. Eur Resusc Council Guidelines 2021: Basic Life Support Resusc. 2021;161(01):98–114. 10.1016/j.resuscitation.2021.02.009. 2021-04-. 10.1016/j.resuscitation.2021.02.009 33773835

[13] Sanfridsson J, Sparrevik J, Hollenberg J, Nordberg P, Djärv T, Ringh M, et al. Drone delivery of an automated external defibrillator – a mixed method simulation study of bystander experience. Scand J Trauma Resusc Emerg Med. 2019;27(40). 10.1186/s13049-019-0622-6. 10.1186/s13049-019-0622-6 PMC6454735 30961651

[14] Zègre-Hemsey JK, Grewe ME, Johnson AM, Arnold E, Cunningham CJ, Bogle BM, et al. Delivery of Automated External Defibrillators via drones in simulated Cardiac arrest: users’ experiences and the Human-Drone Interaction. Resuscitation. 2020;157:83–8. 10.1016/j.resuscitation.2020.10.006. 33080371 10.1016/j.resuscitation.2020.10.006 PMC7769863

[15] Polit D, Beck C. Nursing research: generating and assessing evidence for nursing practice. 10 ed. Philadelphia: Wolters Kluwer; 2017.

[16] DiCicco-Bloom B, Crabtree BF. The qualitative research interview. Med Educ. 2016;40(4):314–21. 10.1111/j.1365-2929.2006.02418.x. 10.1111/j.1365-2929.2006.02418.x 16573666

[17] American Psychological Association. Publication manual of the American Psychological Association: the official guide to APA style. 7 ed. Washington, D.C: American Psychological Association; 2020.

[18] Archibald MM, Ambagtsheer RC, Casey MG, Lawless M. Using zoom videoconferencing for qualitative data collection: perceptions and experiences of researchers and participants. Int J Qual Methods. 2019;18:1–8. 10.1177/1609406919874596.

[19] Elo S, Kyngäs H. The qualitative content analysis process. J Adv Nurs. 2008;62(1): 107 – 15. 10.1111/j.1365-2648.2007.04569.x 10.1111/j.1365-2648.2007.04569.x 18352969

[20] O’Brien BC, Harris IB, Beckman TJ, Reed DA, Cook MD. DA. Standards for Reporting Qualitative Research. A Synthetis of Recommendations. Acad Med. 2014;89(9):1245-51. Doi: 0.1097/ACM.0000000000000388. 10.1097/ACM.0000000000000388 24979285

[21] Holmström IK, Kaminsky E, Lindberg Y, Spangler D, Winblad U. The perspectives of Swedish registered nurses about managing difficult calls to emergency medical dispatch centres: a qualitative descriptive study. BMC Nurs. 2021;9(150). 10.1186/s12912-021-00657-5. 10.1186/s12912-021-00657-5 PMC8371756 34407818

[22] Gustafsson SR, Eriksson I. Quality indicators in telephone nursing – an integrative review. Nurs Open. 2020;8(3):1301–13. 10.1002/nop2.747. 33369230 10.1002/nop2.747 PMC8046143

[23] Allan JL, Farquaharson B, Johnston DW, Jones MC, Choudhary CJ, Johnston M. Stress in telephone helpline nurses is associated with failures of concentration, attention and memory, and with more conservative referral decisions. Br J Psychol. 2013;105(2):200–13. 10.1111/bjop.12030. 24754808 10.1111/bjop.12030

[24] Andersen LW, Holmberg MJ, Berg KM, Donnino MW, Granfeldt A. Hjärtstopp på Sjukhus: en recension. JAMA. 2019;321(12):1200–10. 10.1001/jama.2019.1696. 30912843 10.1001/jama.2019.1696 PMC6482460

[25] Fredman D, Svensson L, Ban Y, Jonsson M, Hollenberg J, Nordberg P et al. Expanding the first link in the chain of survival – Experiences from dispatcher referral of callers to AED locations. Resuscitation. 2016;107(2016-10-01):129 – 34. 10.1016/j.resuscitation.2016.06.022 10.1016/j.resuscitation.2016.06.022 27393179

[26] Ong MEH, Perkins GD, Cariou A. Out-of-hospital cardiac arrest: prehospital management. Lancet. 2018;391(10124):980–8. 10.1016/S0140-6736. 29536862 10.1016/S0140-6736(18)30316-7

[27] Strömsöe A, Afzelius S, Axelsson C, Södersved M, Källestedt L, Enlund M, et al. Improvements in logistics could increase survival after out-of-hospital cardiac arrest in Sweden. J Intern Med. 2013;273(6):622–7. 10.1111/joim.12041. 23360556 10.1111/joim.12041

[28] Valenzuela TD, Roe DJ, Nichol G, Clark LL, Spaite DW, Hardman RG. Outcomes of rapid defibrillation by security officers after cardiac arrest in casinos. N Engl J Med. 2000;343(17):1206–9. 10.1056/NEJM200010263431701. 11071670 10.1056/NEJM200010263431701

[29] Harris P, Kudenchuk J. Cardiopulmonary resuscitation: the science behind the hands. Heart. 2018;104(13):1056–61. 10.1136/heartjnl-2017-312696. 29353251 10.1136/heartjnl-2017-312696

[30] Soar J, Nolan JP, Böttiger BW, Perkins GD, Lott C, Carli P, et al. European resuscitation council guidelines for resuscitation 2015: Sect. 3. Adult advanced life support. Resuscitation. 2015;95:100–47. 10.1016/j.resuscitation.2015.07.016. 26477701 10.1016/j.resuscitation.2015.07.016

[31] Kleinman MK, Brennan EE, Goldberger ZD, Swor RA, Terry M, Bobrow BJ et al. Part 5: Adult Basic Life Support and Cardiopulmonary Resuscitation Quality. 2015 American Heart Association Guidelines Update for Cardiopulmonary Resuscitation and Emergency Cardiovascular Care. Circulation. 2015;132(3):414 – 35. 10.1161/CIR.0000000000000259 10.1161/CIR.0000000000000259 26472993

[32] Pollack RA, Brown SP, Rea T, Aufderheide T, Barbic D, Buick JE, et al. Impact of Bystander Automated External Defibrillator Use on Survival and functional outcomes in shockable observed public Cardiac arrests. Circulation. 2018;137(20):2104–13. 10.1161/CIRCULATIONAHA.117.030700. 29483086 10.1161/CIRCULATIONAHA.117.030700 PMC5953778

[33] Ringh M, Rosenqvist M, Hollenberg J, Jonsson M, Fredman D, Nordberg P, et al. Mobilephone dispatch of laypersons for CPR in out-of-hospital cardiac arrest. N Engl J Med. 2015;372(24):2316–25. 10.1056/NEJMoa1406038. 26061836 10.1056/NEJMoa1406038

[34] Kaminsky E, Lindberg Y, Spangler D, Winblad U, Holmström IK. Registered nurses’ understandings of emergency medical dispatch center work: a qualitative phenomenographic interview study. Nurs Health Sci. 2021;23(2):430–8. 10.1111/nhs.12824. 33665977 10.1111/nhs.12824

[35] Perkins GD, Graesner JT, Semeraro F, Olasveengen T, Soar J, Lott C, et al. European Resuscitation Council guidelines 2021: executive summary. Resuscitation. 2021;161(01):1–60. 10.1016/j.resuscitation.2021.02.003. 2021-04-. 33773824 10.1016/j.resuscitation.2021.02.003

[36] Bergs J, Lambrechts F, Simons P, Vlayen A, Marneffe W, Hellings J, et al. Barriers and facilitators related to the implementation of surgical safety checklists: a systematic review of the qualitative evidence. BMJ Qual Saf. 2015;24(12):776–86. 10.1136/bmjqs-2015-004021. 26199428 10.1136/bmjqs-2015-004021

[37] Golding SE, Horsfield C, Davies A, Egan B, Jones M, Raleigh M, et al. Exploring the psychological health of emergency dispatch centre operatives: a systematic review and narrative synthesis. PeerJ. 2017;5:e3735. 10.7717/peerj.3735. 29062596 10.7717/peerj.3735 PMC5649589

[38] Thomassen Ø, Espeland A, Søfteland E, Lossius HM, Heltne JK, Brattebø G. Implementation of checklists in health care; learning from high-reliability organisations. Scand J Trauma Resusc Emerg Med. 2011;19(53). 10.1186/1757-7241-19-53. 10.1186/1757-7241-19-53 PMC3205016 21967747

[39] World Medical Association. Declaration of Helsinki – Ethical Principles for Medical Research Involving Human Subjects [Internet]. Geneva; World Medical Association. Availible from https://www.wma.net/policies-post/wma-declaration-of-helsinki-ethical-principles-for-medical-research-involving-human-subjects/

